# Insulin signaling is acutely required for long-term memory in *Drosophila*

**DOI:** 10.3389/fncir.2015.00008

**Published:** 2015-03-10

**Authors:** Daniel B. Chambers, Alaura Androschuk, Cory Rosenfelt, Steven Langer, Mark Harding, Francois V. Bolduc

**Affiliations:** ^1^Neuroscience and Mental Health Institute, University of AlbertaEdmonton, AB, Canada; ^2^Department of Pediatrics, University of AlbertaEdmonton, AB, Canada

**Keywords:** learning, long-term memory, insulin receptor, insulin receptor substrate, protein synthesis

## Abstract

Memory formation has been shown recently to be dependent on energy status in *Drosophila*. A well-established energy sensor is the insulin signaling (InS) pathway. Previous studies in various animal models including human have revealed the role of insulin levels in short-term memory but its role in long-term memory remains less clear. We therefore investigated genetically the spatial and temporal role of InS using the olfactory learning and long-term memory model in *Drosophila*. We found that InS is involved in both learning and memory. InS in the mushroom body is required for learning and long-term memory whereas long-term memory specifically is impaired after InS signaling disruption in the ellipsoid body, where it regulates the level of p70s6k, a downstream target of InS and a marker of protein synthesis. Finally, we show also that InS is acutely required for long-term memory formation in adult flies.

## Introduction

Making the right choices is essential to survival. Decisions can be made on intuitive (fast) or rational (slow) thinking (Kahneman, 2011). Slow thinking depends on energetic levels (Gailliot and Baumeister, 2007). Energetic status affects cognition in flies (Hirano et al., 2013; Placais and Preat, 2013) and humans (Gailliot and Baumeister, 2007). Insulin signaling (InS) is an important sensor of nutritional status (Wu et al., 2005) and is highly conserved across species including in *Drosophila* (Petruzzelli et al., 1985; Garofalo, 2002). In addition to its role in normal brain function (reviewed by Ghasemi et al., 2013), InS is also involved in intellectual disability (OMIM 608747, Woods et al., 1996). In addition, InS controls protein synthesis, a key component of long-term memory (Tully et al., 1994), via the phosphorylation of the ribosomal protein S6K. The role of Insulin receptor (InR) and the insulin receptor substrate (IRS- chico in *Drosophila*) in tissue growth is well established (Chen et al., 1996; Bohni et al., 1999). InR affects synaptic density (Chiu and Cline, 2010) and ethanol sensitivity (Corl et al., 2005). Short-term memory defects are seen in C. elegans InR mutants (Lin et al., 2010), in *Drosophila chico* mutants (Naganos et al., 2012) and with pharmacological manipulations in mouse and human (Park et al., 2000; Barros et al., 2001). In addition, InS may regulate metabolism but also neurotransmission via NMDA and GABA signaling (Park et al., 2000). Little is known about the role of InS in protein synthesis dependent long-term memory and its acute role in adults. We used the *Drosophila* olfactory conditioning paradigm (Tully and Quinn, 1985) where protein synthesis dependent and independent memory can easily be measured to test the spatio-temporal requirement for InS in learning and memory.

## Methods

### *Drosophila* strains

*Drosophila* stocks were maintained at 22°C on cornmeal agar medium. University of Alberta ethics and biohazard regulations were observed in the laboratory. *Drosophila* UAS-chico RNAi and UAS-dInR RNAi lines were obtained from the Vienna *Drosophila* RNAi Center (VDRC) and are labeled by their accession number. Wild-type, OK107GAL4, ELAVGAL4, FEB170GAL4, and 232GAL4 were obtained from Dr. Tim Tully (Cold Spring Harbor Laboratory). UAS-Chico was obtained from Dr. Minoru Saitoe. Insulin DN is from Bloomington Stock center (8252). All stocks were homogenized with the WT background [w^1118^ (isoCJ1)] by backcrossing for 6 generations.

### Genetic crosses

In all cases virgin females of the GAL4 driver or wild type flies were collected and crossed to the UAS-transgene males.

### Pavlovian olfactory conditioning

*Drosophila* were raised after being dechorionated at 22-23°C. Adult flies 1-3 days old were used for the behavior experiments. Flies were trained as described previously for learning and memory (Tully et al., 1994). About 100 flies are aspirated for each experiment. Flies are placed inside a tube containing an electrified grid.

As described before, flies are given 90 s to acclimate and then exposed sequentially to 3-octanol (OCT) and 4-methylcyclohexanol (MCH) with relative concentration adjusted for equal averseness. A footshock is paired for 60 s with either other and then the paired odor is reversed in the subsequent trial. The shock consists of twelve 1.25 s pulses of 60 V DC with 5 s interpulse interval. The flies are provided with fresh air between the 2 odors for 45 s. After giving the second odor, the flies are again given 45 of fresh air before being moved to the elevator. 90 s later the flies are brought to the choice point where they are exposed on 1 side with the odor that was paired with the shock and on the other with the odor not paired with the shock. Flies are given 2 min to do their choice before being trapped and counted. For learning, a single training session is used. For spaced training, the flies receive the same training session without the testing part but it is repeated 10 times with 15 min rest interval between each training session. Flies are allowed to rest for 24 h and tested after that. For massed training, flies are given 10 training sessions also but this time without rest interval. For each performance index (PI), there is a component of shocking to each odor which allows to balance any slight odor preference. The PI is calculated as the average fraction of the number of flies avoiding the shocked odor minus the fraction of flies avoiding the non-shocked odor. So, if flies distribute 50:50, the PI would be zero.

For experiments where multiple groups are present, a One-Way ANOVA followed by a Tukey post test was performed. For experiments where 2 groups are compared, Student *T*-test was used. Statistics were performed with Prism and JMP. Comparison with one asterisk are significant to *P* < 0.05, two asterisks indicate *P* < 0.001 and three asterisks *P* < 0.0001. All graphs depict ± s.e.m.

### Task-relevant sensory controls and motor controls

To make sure that the observed defects were not related to lack of olfaction or shock sensation or impairment with locomotion, the flies were tested for those modalities as well. Olfactory acuity and shock reactivity were assessed as in Boynton and Tully (1992) and Dura et al. (1993) For odor avoidance, naïve flies were put in the t-maze and given the choice between the odor used in the experiment (OCT or MCH) or air. The odor being naturally aversive to the flies, most flies avoided it. The flies were given 2 min to choose before being trapped and counted. The side of the odor and air was alternated between trials to avoid odor build up. For shock reactivity, a similar experiment was conducted but this time with collection tubes containing grid being placed on each side of the t-maze. The flies were brought to the choice point and then the shock was turned on.

### Conditional transgenic expression

Acute heat-shock was performed as in Yin et al. (1994) and more recently used to show the acute role of dFMRP in memory (Bolduc et al., 2008). Flies were grown in a separate chamber at 18°C to minimize the leaky expression of the HSP70GAL4 driver. Adult flies were transferred to fresh bottles and placed overnight at 23°C. Heat shock was performed the next morning by placing the flies in a circulating water bath at 37°C for 35 min. Flies were then transferred to regular food vials and let to rest for 3 h at 25°C. The flies were then trained as usual at 25°C.

### Immunohistochemistry

One to three days old female flies were dissected and processed as described previously. Bolduc et al. (2010) Flies are dissected in PBS and then transferred to 4%PFA for 10 min at room temperature. Flies are placed in vacuum for 1 h in 0.25% Triton 4%PFA. The flies were then transferred to penetration/blocking buffer for 2 h at 4°C on a rotating plate. Brains are transferred to primary antibody and incubated overnight on a rotating plate at 4°C. The next day, flies are washed 3 times in wash buffer for 10 min. Brains are then transferred in secondary antibody and incubated overnight. On the third day, brains are washed again 3 times and mounted. Imaging was done using a 20 × objective on Zeiss LSM700 and processed with the Zeiss ZEN 2009 software. There was no processing of the images.

For FAS-II staining, we used 1d4 antibody (1:200) from Developmental Hybridoma study bank. We used the midline crossing phenotype established by Michel et al as reference. For p70S6K Thr398 we used Cell signaling (9209S) at a concentration of 1:25. All images within a given panel were acquired with the same gain to allow for comparison. Quantification was performed using ImaJ. The brain region was selected for the measurements. We used Cy3 anti-mouse as secondary antibody (Jackson Immuno Laboratory) at concentration of 1:200.

For the experiments with training, flies were trained as usual to MCH. In the untrained group, flies were presented with the odors as usual but without receiving the shock paired to the odors. Imaging was performed at 1 day after spaced training or mock spaced training.

## Results

**Figure 1:**
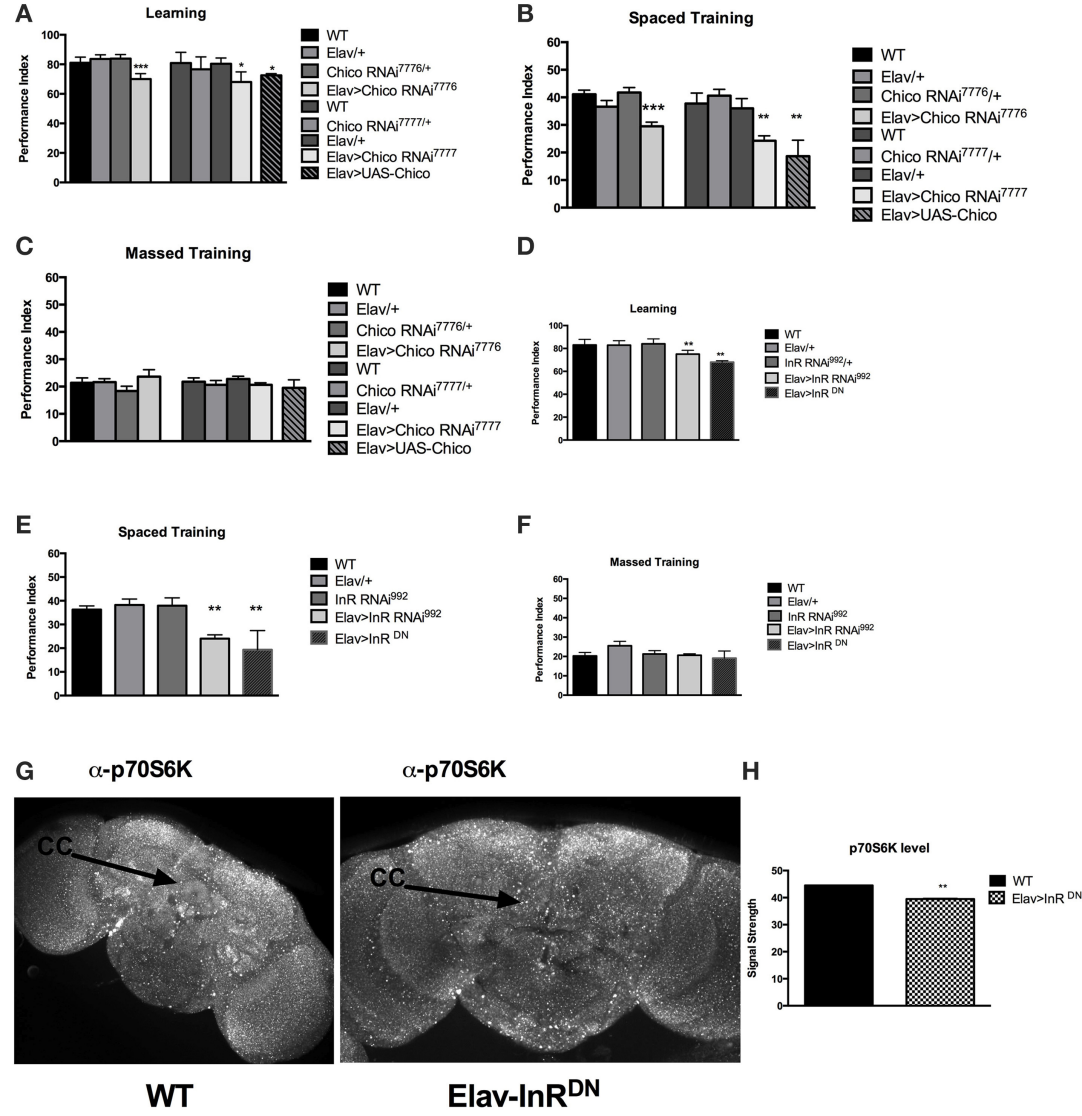
**Pan-neuronal disruption of Insulin signaling results in memory defects**. **(A)** Learning is significantly defective in *Drosophila* expressing UAS-RNAi against the insulin receptor substrate, chico pan-neuronally. We expressed 2 different RNAi constructs: [Elav>ChicoRNAi^7776^] (*P* = 0.005, *N* = 6 PI per genotype) and [Elav>ChicoRNAi^7777^] (*P* = 0.0104, *N* = 6 PI per genotype). In addition, pan-neuronal overexpression of chico [Elav>UAS-chico] led to a significant (*P* = 0.0015, *N* = 8 PI per genotype) defect in learning. The genetic controls ElavGAL4 [Elav/+], chicoRNAi [chicoRNAi^7776^], [chicoRNAi^7777^] and InRRNAi [InRRNAi^992^] show no defects compared to wild-type flies [WT]. **(B)** 1-day memory after spaced training is impaired by loss or gain of function of chico in neurons. RNAi against chico resulted in significant defect in 1 day memory for both constructs [Elav>ChicoRNAi^7776^] (*P* < 0.00001, *N* = 8 PI per genotype); [Elav>ChicoRNAi^7777^] (*P* < 0.00001, *N* = 8 PI per genotype). Overexpression of chico [Elav>UAS-chico] also resulted in significant defect in 1 day memory (*P* = 0.0004, *N* = 8 PI per genotype) **(C)** No significant defects in 1 day memory after massed training were observed between Chico RNAi or UAS-chico expressing flies compared to genetic controls (*N* = 8 PI per genotype). **(D)** Similarly, pan-neuronal disruption of Insulin receptor leads to learning defects either via expression of UAS-InR RNAi [Elav>InR RNAi^992^] (*P* = 0.0082, *N* = 4 PI per genotype) or with the expression of a dominant negative InR [Elav>InR^DN^] (*P* < 0.00001, *N* = 4 PI per genotype). There was no significant defect in any of the genetic appropriate controls. **(E)** Significant defects in 1 day memory after spaced training were also observed in transgenic flies expressing [Elav>InR RNAi^992^] (*P* < 0.00001, *N* = 8 PI per genotype) or [Elav>InR^DN^] (*P* = 0.0052, *N* = 8 PI per genotype). **(F)** No significant defects were seen in flies expressing InR RNAi or DN or in the appropriate genetic controls. **(G)** Representative level of p70S6K in the brain of WT and flies expressing InR^DN^ pan-neuronally [Elav>InR^DN^]. **(H)** Significant decreased level of p70S6K is observed in Elav>InR^DN^ flies (*N* = 5 brains per genotype, *P* = 0.0022) All graphs depict mean ± s.e.m. ^*^p < 0.05; ^**^p < 0.001; ^***^p < 0.0001.

**Figure 2:**
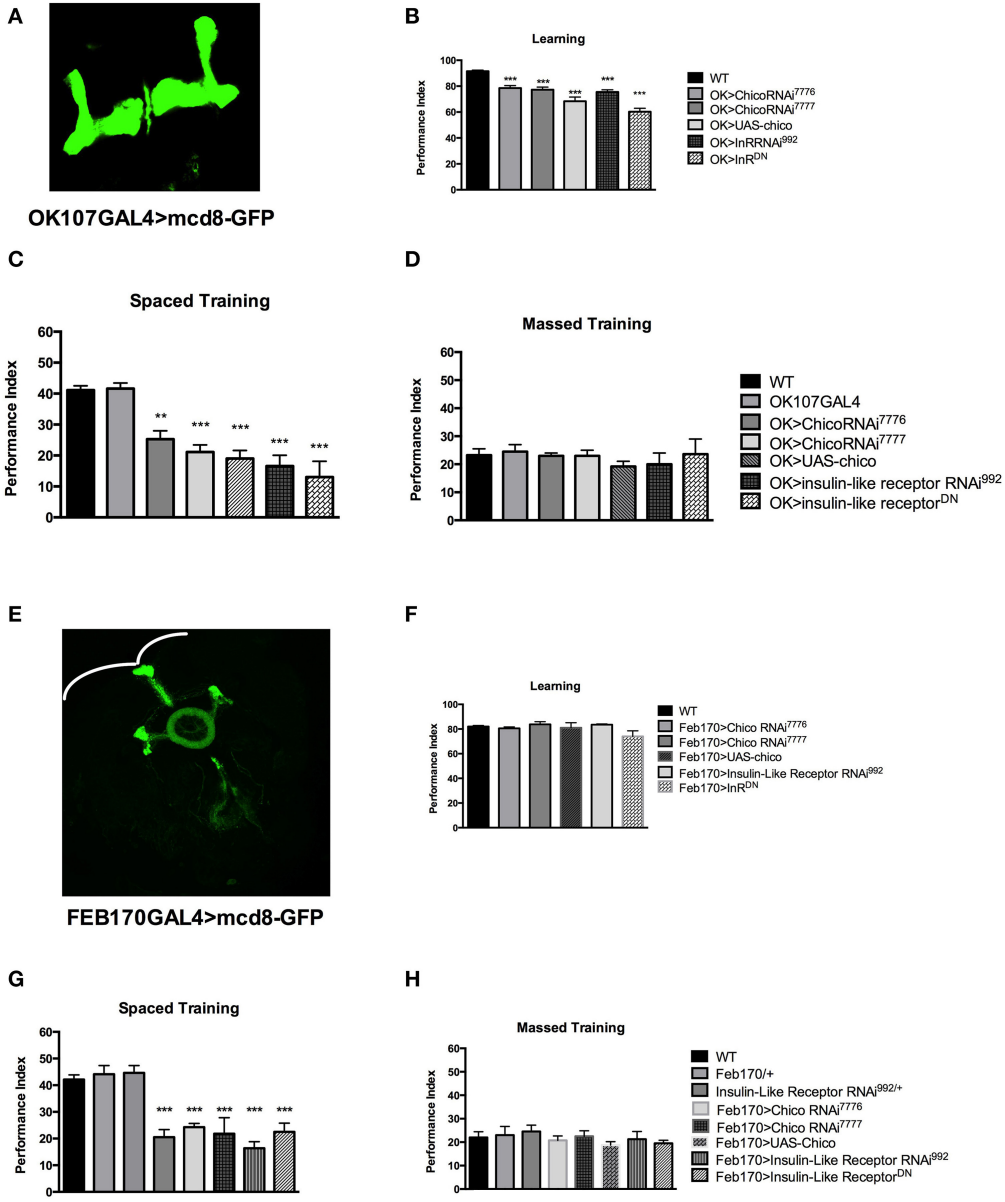
**Insulin signaling participates in different stages of memory formation**. **(A)** Expression pattern of OK107GAL4. **(B)** Learning is defective with mushroom body (MB) expression of chico RNAi [OK>chicoRNAi^7776^] (*P* < 0.05, *N* = 4 PI); [OK>chicoRNAi^7777^] (*P* < 0.05, *N* = 4 PI) or overexpression of chico [OK>UAS-chico] (*P* < 0.0001, *N* = 6 PI per genotype). Similarly expression of InRRNAi [OK>InRRNAi^992^] (*P* < 0.001, *N* = 4 PI) and InR^DN^ [OK>InR^DN^] (*P* < 0.0001, *N* = 4 PI per genotype) leads to learning defects. **(C)** Significant defects in 1 day memory after spaced training are observed with MB expression of chicoRNAi [OK>chicoRNAi^7776^] (*P* < 0.001, *N* = 8 PI per genotype), [OK>chicoRNAi^7777^] (*P* < 0.00001, *N* = 8 PI per genotype) or chico overexpression [OK>UAS-chico] (*P* < 0.0001, *N* = 6 PI per genotype). Similarly, mushroom body expression of InR RNAi^992^ [OK>InRRNAi^992^] (*P* < 0.00001, *N* = 8 PI per genotype) or InR^DN^ [OK>InR^DN^] (*P* < 0.00001, *N* = 6 PI per genotype) leads to significant memory defects. **(D)** No defect is seen in 1-day memory after massed training for the same genotypes. **(E)** Expression pattern of the FEB170GAL4 illustrated by expressing mCD8::GFP. **(F)** No significant defect is seen with FEB170 expression of chico RNAi or overexpression as well as with InR RNAi or InR^DN^ in learning (*N* = 4 PI per genotype). **(G)** 1-day memory after spaced training is defective in flies expressing chicoRNAi [Feb>chicoRNAi^7776^] (*P* < 0.001, *N* = 8 PI per genotype) [Feb>chicoRNAi^7777^] (*P* < 0.0001, *N* = 8 PI) or with chico overexpression [Feb>UAS-chico] (*P* < 0.0001, *N* = 8 PI per genotype). Similarly 1-day memory is defective after spaced training with the expression of InR RNAi^992^ [Feb170>InR RNAi^992^] (*P* < 0.0001, *N* = 8 PI per genotype) or InR^DN^ [Feb>InR^DN^] (*P* < 0.0001, *N* = 8 PI) in the central complex. **(H)** No significant defect is seen in the same genotypes in 1-day memory after massed training. All graphs depict mean ± s.e.m. ^**^p < 0.001; ^***^p < 0.0001.

Considering the previous evidence of learning defect in the *chico* mutant flies, we sought to see if long-term memory was also affected by InS. We started by focusing on chico, the only *Drosophila* homolog of the insulin receptor substrate (IRS). We first expressed RNAi against the *Drosophila* insulin receptor substrate, chico, pan-neuronally using ElavGAL4. We were able to replicate the previous findings from Naganos et al. (2012) showing a requirement of chico in learning, using two different transgenic RNA interference (RNAi) lines against chico. Indeed, learning (WT vs. Elav>chico RNAi^7776^, *P* = 0.005; WT vs. Elav>chico RNAi^7777^, *P* = 0.0104) was significantly defective. Moreover, we found that long-term memory (WT vs. Elav>chico RNAi^7776^, *P* < 0.0001; WT vs. Elav>chico RNAi^7777^, *P* = 0.0067) was also significantly affected (Figures 1A,B) but that 1 day-memory after massed training was intact (Figure 2C). Since, only spaced training can lead to protein synthesis, these findings suggests that InS is specifically involved in protein synthesis dependent memory at 1-day. We also observed that chico overexpression led to significant defects in learning (WT vs. UAS-chico, *P* = 0.0015) and 1-day memory after spaced training (WT vs. UAS-chico, *P* = 0.0004) (Figures 1A,B) while 1-day memory after massed training intact (ANOVA, *P* = 0.5364) (Figure 1C). Similar findings of memory defects in context of loss-of-function or overexpression have been observed in our *Drosophila* model before for Fragile X mutant flies (Bolduc et al., 2008) but also clinically in intellectual disability syndromes such as Fragile X (Vengoechea et al., 2012) and Rett syndrome (Lugtenberg et al., 2009). Control experiments for shock and odor sensation did not reveal any significant defect (Supplementary Figures 1A–D). Next we tested the effect of pan-neuronal disruption of the Insulin-like receptor (InR) using RNAi and dominant negative (DN) transgenic flies. We observed that pan-neuronal expression of InR RNAi led to a significant defect in learning (WT vs. Elav>InR^992^, *P* = 0.0082) (Figure 1D). Similarly, expression of a dominant negative (DN) form of the dInR led to significant defect in learning (WT vs. Elav>InR^DN^, *P* < 0.0001) Next, we tested the performance at 1 day after spaced training of the InR RNAi or DN expressing flies and observed a significant defect in long-term memory for both (WT vs. Elav>InRRNAi^992^, *P* < 0.0001; WT vs. Elav>InR^DN^, *P* = 0.0052) (Figure 1E). The same transgenic flies did not present any defect at 1 day after massed training (ANOVA, *P* = 0.1593) (Figure 1F). There were no olfactory or shock sensory defects (Supplementary Figures 1E,F). In addition, considering that learning defects can be caused by gross malformation of the mushroom bodies, we imaged the mushroom bodies using FAS II antibody. There was no evidence of mushroom body crossing over (Supplementary Figure 1G). We found that p70S6K signal, a downstream target of InS and marker of protein synthesis, is significantly reduced after pan-neuronal expression of InR^DN^. We compared the level of protein synthesis in WT and Elav>InR^DN^ brains and observed that flies expressing pan-neuronally InR^DN^ had significantly decreased overall level of p70S6K, a downstream target of the InS pathway and a marker of AKT dependent protein synthesis (*P* = 0.0022). In addition, we noted that the basal level of protein synthesis in the ellipsoid body was markedly decreased compared to control flies (Figure 1G). Overexpression of chico led to increased size of puncta and loss of homogenous signal in the central complex (Supplementary Figure 1H) In summary, both Insulin receptor and insulin receptor substrate are involved in learning and 1-day memory after spaced training (protein synthesis dependent memory) but not in 1-day after massed training.

Next, we investigated the spatial requirement of InR and Chico in learning and memory within the fly brain. Naganos et al. (2012) was able to rescue the *chico* mutants learning defect by expressing chico in the mushroom bodies. We therefore started by testing the effect of insulin receptor (InR) and chico disruption in the mushroom bodies (using the OK107 GAL4 driver-with a wide MB expression Figure 2A). We observed that mushroom body expression of the UAS-chicoRNAi^7776^, UAS-chicoRNAi^7777^ or UAS-chico lead to significant defect in learning (WT vs. OK>chicoRNAi^7776^, *P* = 0.001; WT vs. chicoRNAi^7777^, *P* < 0.0001; WT vs. OK>UAS-chico, *P* < 0.0001) (Figure 2B). Similarly, mushroom body expression of UAS-InR RNAi^992^ or UAS-InR^DN^ (WT vs. OK>InR RNAi^992^, *P* < 0.0001; WT vs. OK>InR^DN^, *P* < 0.0001) also led to significant learning defects. Memory after spaced training was also impaired with disruption of either chico or InR (WT vs. OK>chicoRNAi^7776^, *P* = 0.0001; WT vs. OK>chicoRNAi^7777^, *P* < 0.00001; WT vs. OK>UAS-chico, *P* < 0.00001; WT vs. OK>InRRNAi^992^, *P* < 0.00001; WT vs. OK>InR^DN^, *P* < 0.00001) but had no effect on memory after massed training (ANOVA, 0.9451) (Figures 2C,D) or sensory controls (Supplementary Figures 2A,B).

Considering the difference in p70S6K signal between WT and Elav>InR^DN^ in the central complex (Figure 1G), we decided to investigate the role of InS in the central complex. We tested the effect of expression of the InR RNAi, InR^DN^ and chico overexpression and RNAi in the central complex- ellipsoid body and possibly the noduli (using FEB170 GAL4 driver that localized NMDAR for LTM Wu et al., 2007) (Figure 2E). We found that learning was not significantly affected when compared to wild-type flies (ANOVA 0.3431) but that 1-day memory after spaced training was significantly affected with disruption of chico (WT vs. Feb170>chicoRNAi^7776^, *P* < 0.0001; WT vs. Feb170>chicoRNAi^7777^, *P* < 0.0001; or WT vs. FEB>UAS-chico, *P* = 0.0049) or InR (Feb170>InRRNAi^992^, *P* < 0.00001; Feb170>InR^DN^, *P* < 0.0001) (Figures 2F,G). One-day memory after massed training was normal in all groups (ANOVA, 0.6365) (Figure 2H). The transgenic flies responded normally to shock and olfaction. (Supplementary Figures 2C,D). Expression in another sub-region of the central complex (Supplementary Figure 3) did not lead to memory defect. These results suggest that learning and by consequence memory may be affected when disrupting InS in the MB but that protein synthesis dependent memory related to InS is controlled by InS in the central complex. Other pathways control protein synthesis as the level of protein synthesis, as tested with p70S6K is diminished but not abolished with one of the strongest construct, Elav>InR^DN^.

**Figure 3:**
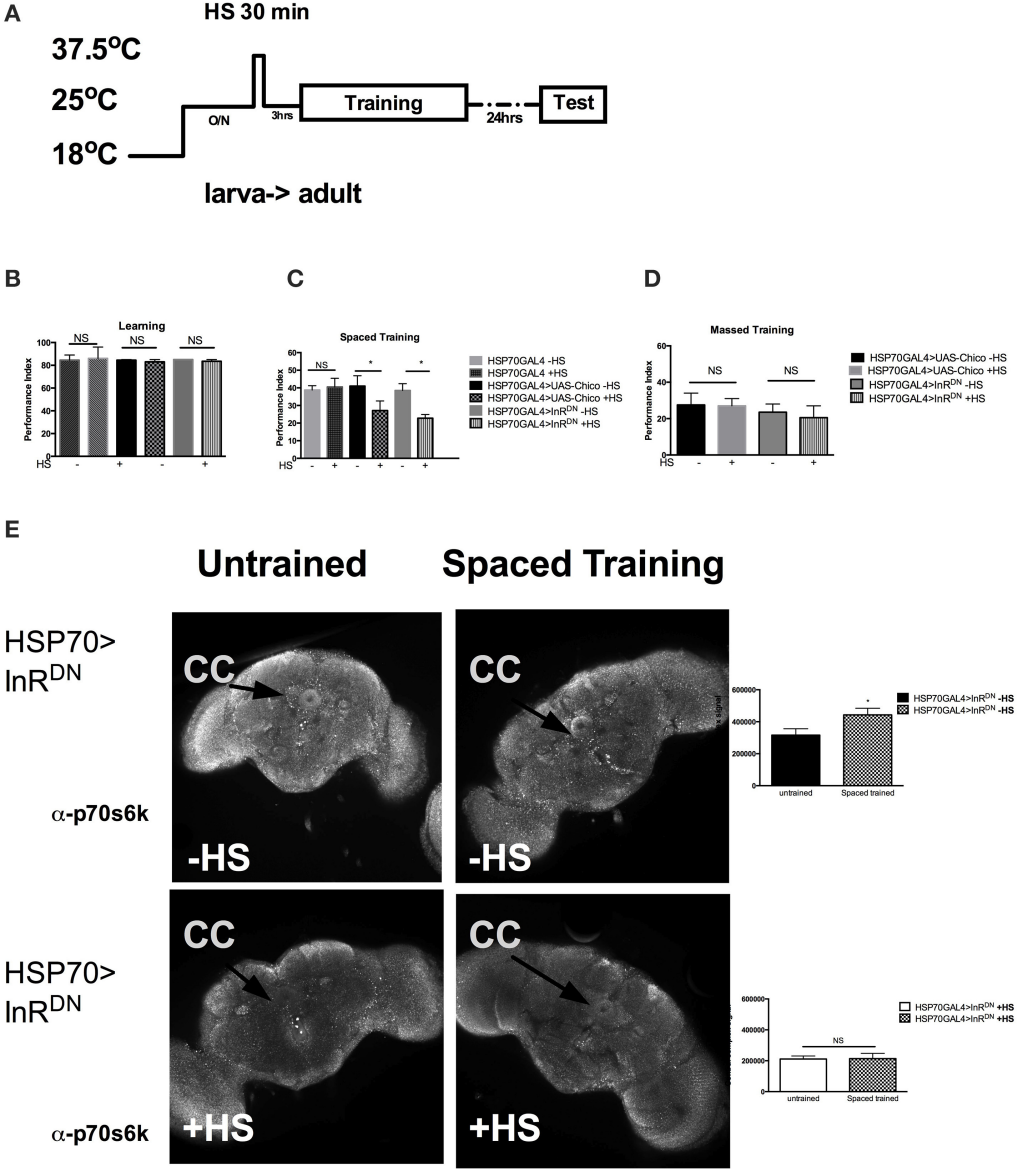
**Insulin signaling is acutely required for memory formation**. **(A)** Protocol used to express the transgenes acutely in adult. The protocol depicted is for 1 day memory after spaced training. For learning, the testing immediately follows training. **(B)** Acute expression of UAS-chico [HSP70GAL4>UAS-chico +HS] (*P* = 0.5425) or InR^DN^ [HSP70GAL4>InR^DN^ +HS] (*P* = 0.4226, *N* = 2 PI per genotype) does not affect learning. **(C)** Acute expression of UAS-chico [HSP70GAL4>UAS-chico +HS] (*P* = 0.0316, *N* = 8 PI per genotype) or InR^DN^ [HSP70GAL4>InR^DN^ +HS] (*P* = 0.0069, *N* = 8 PI per genotype) disrupts memory after spaced training. **(D)** No defect is seen after the same treatment in 1 day memory after massed traininig (*P* > 0.05, *N* = 2 PI per groups). **(E)** The first row shows representative brains immunohistochemistry with p70s6k from HSP70GAL4>InR^DN^—**HS** flies with mock training (presented with odors but no shock) vs. spaced training. The second row shows representative brain immunohistochemistry with p70s6k from HSP70GAL4>InR^DN^ **+HS** with mock vs. spaced training. Quantification of the signal strength in the central complex shows a significant (*P* = 0.0351, *N* = 4 biological replicates per group) increase after spaced training compared to mock training in HSP70>InR^DN^ flies only when not exposed to heat shock. All graphs depict mean ± s.e.m. ^*^p < 0.05.

Next we investigated if InS was required acutely in adult for memory formation. We utilized the HSP70-GAL4 driver P26 to express acutely in adult flies the UAS-dInR^DN^ or the UAS-chico transgene. As shown previously, a very brief 35 min heat shock (HS) is sufficient to trigger transient expression of HSP70GAL4 (Figure 3A) that peaks at 3 h and decline after 6 h (Yin et al., 1994). Interestingly, acute expression of neither the InR^DN^ (−HS vs. +HS, *P* = 0.5425) nor chico (−HS vs. +HS, *P* = 0.4226) led to defects in immediate learning (Figure 3B). However, we found a significant defect in 1-day memory after spaced training in flies expressing the UAS-InR^DN^ (−HS vs. +HS, *P* = 0.0069) or the UAS-chico (−HS vs. +HS, *P* = 0.0316) when compared to flies without expression (Figure 3C). No defect was observed in 1-day memory after massed training with the same heat-shock protocol. These results suggest that InS is required acutely in memory formation. Next, we observed that the level of p-S6K, a downstream target of InS and a marker of protein synthesis, was increased after spaced training in the central complex of HSP70>InR^DN^ of flies that did not receive heat shock but was significantly decreased and failed to rise with spaced training in the group of flies that received spaced training (Figure 3D). The untrained flies still received the odors but did not receive the footshock included in the training protocol.

## Discussion

The brain has the highest energy requirement of all organs, reaching 87% of the resting metabolic rate in childhood (Kuzawa et al., 2014). Insulin signaling (InS) is a key component in the cellular management of energy but its role in long-term memory formation remains largely unknown. Considering the emerging importance of *Drosophila* models of memory in understanding intellectual disability and cognitive function, we sought to understand the role of InS in memory formation, more specifically protein synthesis dependent memory. Our study shows that two key components, the insulin receptor (InR) and the insulin receptor substrate (chico) are required for normal learning and protein synthesis dependent memory but not for protein synthesis independent memory. We show that InS in the ellipsoid body, a region highly connected and compared to the basal ganglia (Lin et al., 2013), is required for long-term memory and is one of the site of protein synthesis in response to spaced training. Other brain regions show increased p70S6K activity and may be related to protein synthesis secondary to other metabolic cascades. Interestingly, InS sustains different stage of memory formation in different regions of the brain, as was shown for NMDAR (Wu et al., 2007). Finally, we show that acute InS disruption affects protein synthesis dependent memory at 1 day. This is important as it shows potential for acute rescue of protein synthesis defect via the targeting of InS. Future experiments could determine more specifically when during training and consolidation the role of inS is required.

## Author contributions

DC and FB conceived and designed the project and analyzed the experiments. DC, FB, SL, and CR performed the behavior experiments. DC, AA, and FB wrote the paper with comments from the other authors. AA and MH performed the immunohistochemistry experiments.

### Conflict of interest statement

The authors declare that the research was conducted in the absence of any commercial or financial relationships that could be construed as a potential conflict of interest.

## References

[B1] Barros D. M. Mello e Souza T. de Souza M. M. Choi H. DeDavid e Silva T. Lenz G. (2001). LY294002, an inhibitor of phosphoinositide 3-kinase given into rat hippocampus impairs acquisition, consolidation and retrieval of memory for one-trial step-down inhibitory avoidance. Behav. Pharmacol. 12, 629–634 10.1097/00008877-200112000-00007 11856900

[B2] Bohni R. Riesgo-Escovar J. Oldham S. Brogiolo W. Stocker H. Andruss B. F.. (1999). Autonomous control of cell and organ size by CHICO, a Drosophila homolog of vertebrate IRS1-4. Cell 97, 865–875. 10.1016/S0092-8674(00)80799-0 10399915

[B3] Bolduc F. V. Bell K. Cox H. Broadie K. S. Tully T. (2008). Excess protein synthesis in Drosophila fragile X mutants impairs long-term memory. Nat. Neurosci. 11, 1143–1145. 10.1038/nn.2175 18776892 PMC3038669

[B4] Bolduc F. V. Bell K. Rosenfelt C. Cox H. Tully T. (2010). Fragile x mental retardation 1 and filamin a interact genetically in Drosophila long-term memory. Front. Neural Circuits 3:22. 10.3389/neuro.04.022 20190856 PMC2813723

[B5] Boynton S. Tully T. (1992). latheo, a new gene involved in associative learning and memory in Drosophila melanogaster, identified from P element mutagenesis. Genetics 131, 655–672. 1321066 10.1093/genetics/131.3.655 PMC1205037

[B6] Chen C. Jack J. Garofalo R. S. (1996). The Drosophila insulin receptor is required for normal growth. Endocrinology 137, 846–856. 8603594 10.1210/endo.137.3.8603594

[B7] Chiu S. L. Cline H. T. (2010). Insulin receptor signaling in the development of neuronal structure and function. Neural Dev. 5:7. 10.1186/1749-8104-5-7 20230616 PMC2843688

[B8] Corl A. B. Rodan A. R. Heberlein U. (2005). Insulin signaling in the nervous system regulates ethanol intoxication in Drosophila melanogaster. Nat. Neurosci. 8, 18–19. 10.1038/nn1363 15592467

[B9] Dura J. M. Preat T. Tully T. (1993). Identification of linotte, a new gene affecting learning and memory in Drosophila melanogaster. J. Neurogenet. 9, 1–14. 10.3109/01677069309167272 8295074

[B10] Gailliot M. T. Baumeister R. F. (2007). The physiology of willpower: linking blood glucose to self-control. Pers. Soc. Psychol. Rev. 11, 303–327. 10.1177/1088868307303030 18453466

[B11] Garofalo R. S. (2002). Genetic analysis of insulin signaling in Drosophila. Trends Endocrinol. Metab. 13, 156–162. 10.1016/S1043-2760(01)00548-3 11943559

[B12] Ghasemi R. Haeri A. Dargahi L. Mohamed Z. Ahmadiani A. (2013). Insulin in the brain: sources, localization and functions. Mol. Neurobiol. 47, 145–171. 10.1007/s12035-012-8339-9 22956272

[B13] Hirano Y. Masuda T. Naganos S. Matsuno M. Ueno K. Miyashita T.. (2013). Fasting launches CRTC to facilitate long-term memory formation in Drosophila. Science 339, 443–446. 10.1126/science.1227170 23349290

[B14] Kahneman D. (2011). Thinking, Fast and Slow. New York, NY: Farrar, Straus and Giroux.

[B15] Kuzawa C. W. Chugani H. T. Grossman L. I. Lipovich L. Muzik O. Hof P. R.. (2014). Metabolic costs and evolutionary implications of human brain development. Proc. Natl. Acad. Sci. U.S.A. 111, 13010–13015. 10.1073/pnas.1323099111 25157149 PMC4246958

[B16] Lin C. H. Tomioka M. Pereira S. Sellings L. Iino Y. van der Kooy D. (2010). Insulin signaling plays a dual role in Caenorhabditis elegans memory acquisition and memory retrieval. J. Neurosci. 30, 8001–8011. 10.1523/JNEUROSCI.4636-09.2010 20534848 PMC6632696

[B17] Lin C. Y. Chuang C. C. Hua T. E. Chen C. C. Dickson B. J. Greenspan R. J.. (2013). A comprehensive wiring diagram of the protocerebral bridge for visual information processing in the Drosophila brain. Cell Rep. 3, 1739–1753. 10.1016/j.celrep.2013.04.022 23707064

[B18] Lugtenberg D. Kleefstra T. Oudakker A. R. Nillesen W. M. Yntema H. G. Tzschach A.. (2009). Structural variation in Xq28: MECP2 duplications in 1% of patients with unexplained XLMR and in 2% of male patients with severe encephalopathy. Eur. J. Hum. Genet. 17, 444–453. 10.1038/ejhg.2008.208 18985075 PMC2986218

[B19] Naganos S. Horiuchi J. Saitoe M. (2012). Mutations in the Drosophila insulin receptor substrate, CHICO, impair olfactory associative learning. Neurosci. Res. 73, 49–55. 10.1016/j.neures.2012.02.001 22342328

[B20] Park C. R. Seeley R. J. Craft S. Woods S. C. (2000). Intracerebroventricular insulin enhances memory in a passive-avoidance task. Physiol. Behav. 68, 509–514. 10.1016/S0031-9384(99)00220-6 10713291

[B21] Petruzzelli L. Herrera R. Garcia-Arenas R. Rosen O. M. (1985). Acquisition of insulin-dependent protein tyrosine kinase activity during Drosophila embryogenesis. J. Biol. Chem. 260, 16072–16075. 3934169

[B22] Placais P. Y. Preat T. (2013). To favor survival under food shortage, the brain disables costly memory. Science 339, 440–442. 10.1126/science.1226018 23349289

[B23] Tully T. Preat T. Boynton S. C. Del Vecchio M. (1994). Genetic dissection of consolidated memory in Drosophila. Cell 79, 35–47. 10.1016/0092-8674(94)90398-0 7923375

[B24] Tully T. Quinn W. G. (1985). Classical conditioning and retention in normal and mutant Drosophila melanogaster. J. Comp. Physiol. A 157, 263–277. 10.1007/BF01350033 3939242

[B25] Vengoechea J. Parikh A. S. Zhang S. Tassone F. (2012). *De novo* microduplication of the FMR1 gene in a patient with developmental delay, epilepsy and hyperactivity. Euro. J. Hum. Genet. 20, 1197–1200. 10.1038/ejhg.2012.78 22549406 PMC3476717

[B26] Woods K. A. Camacho-Hubner C. Savage M. O. Clark A. J. (1996). Intrauterine growth retardation and postnatal growth failure associated with deletion of the insulin-like growth factor I gene. N. Engl. J. Med. 335, 1363–1367. 10.1056/NEJM199610313351805 8857020

[B27] Wu C. L. Xia S. Fu T. F. Wang H. Chen Y. H. Leong D.. (2007). Specific requirement of NMDA receptors for long-term memory consolidation in Drosophila ellipsoid body. Nat. Neurosci. 10, 1578–1586. 10.1038/nn2005 17982450 PMC3055246

[B28] Wu Q. Zhang Y. Xu J. Shen P. (2005). Regulation of hunger-driven behaviors by neural ribosomal S6 kinase in drosophila. Proc. Natl. Acad. Sci. U.S.A. 102, 13289–13294. 10.1073/pnas.0501914102 16150727 PMC1201572

[B29] Yin J. C. Wallach J. S. Del Vecchio M. Wilder E. L. Zhou H. Quinn W. G.. (1994). Induction of a dominant negative CREB transgene specifically blocks long-term memory in Drosophila. Cell 79, 49–58. 10.1016/0092-8674(94)90399-9 7923376

