# Supplementary figures and images for: Insulin signaling is acutely required for long-term memory in Drosophila
Source: Front Neural Circuits. 2015 Mar 10;9:8. doi: 10.3389/fncir.2015.00008 (PMC4354381; doi:10.3389/fncir.2015.00008)

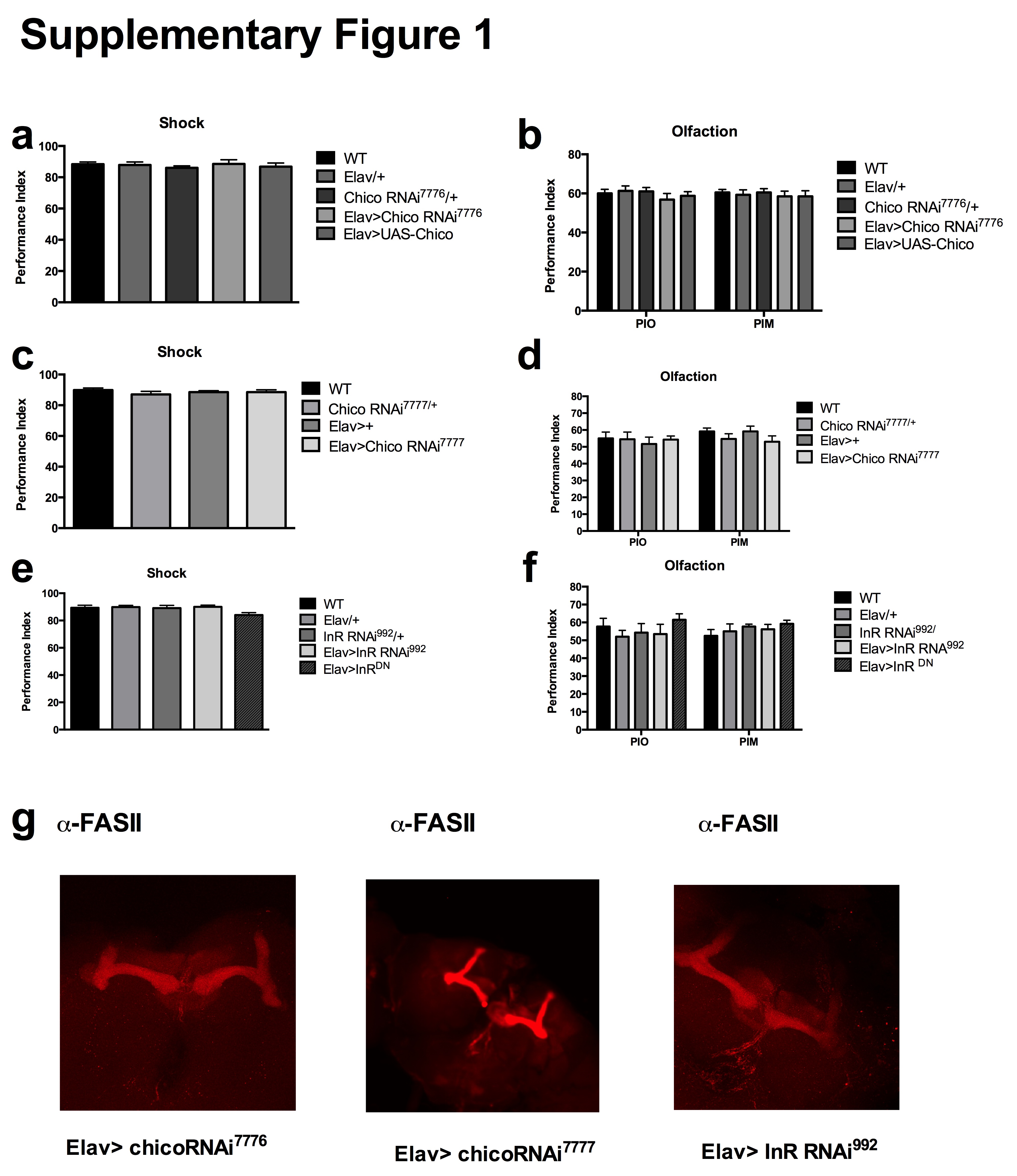

Supplement: Supplementary file 1 [file Image1.JPEG]

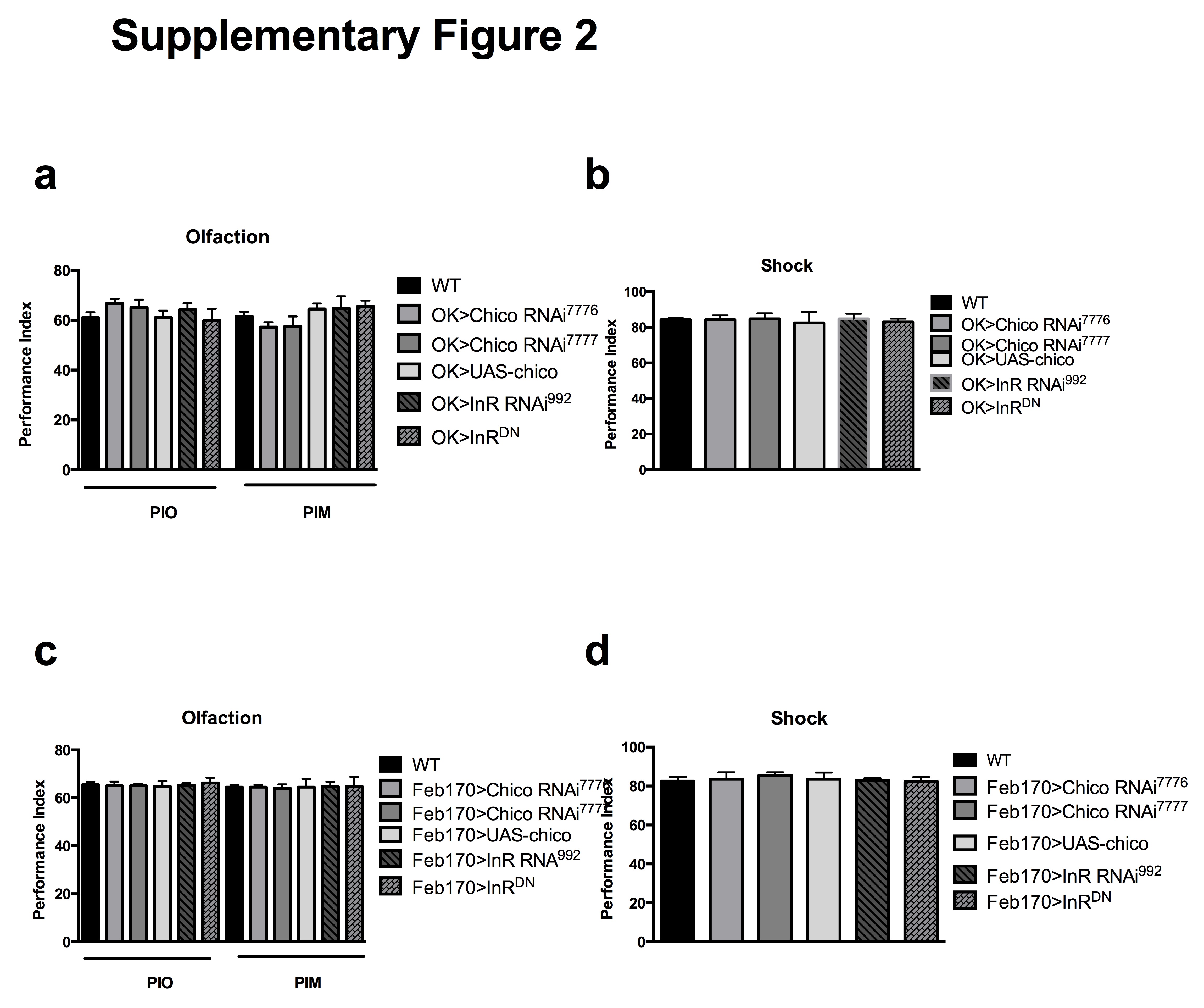

Supplement: Supplementary file 2 [file Image2.JPEG]

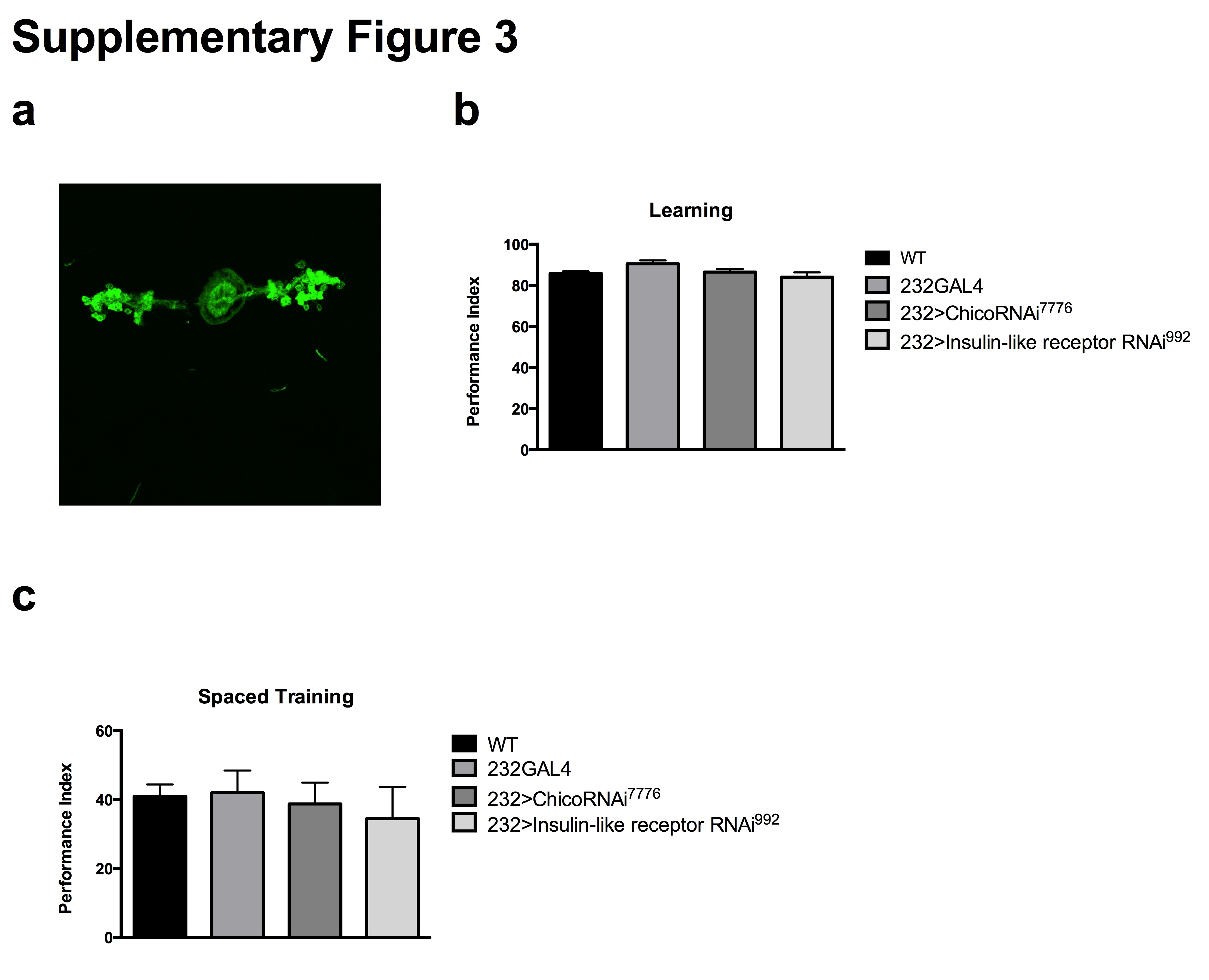

Supplement: Supplementary file 3 [file Image3.JPEG]
