# Supplementary material for: Insulin signaling is acutely required for long-term memory in Drosophila
Source: Front Neural Circuits. 2015 Mar 10;9:8. doi: 10.3389/fncir.2015.00008 (PMC4354381; doi:10.3389/fncir.2015.00008)
Supplement: Supplementary file 4 [file DataSheet1.DOCX]

SUPPLEMENTARY MATERIAL FOR

**Insulin signaling is acutely required for long-term memory in Drosophila**

Daniel B Chambers^2^, Alaura Androschuk^1^, Cory Rosenfelt^1^, Steven Langer^1^, , Mark Harding^1^ and Francois V Bolduc^1, 2^*

**Supplementary Figure Legends**

**Supplementary Figure 1.** Task relevant sensory controls for pan-neuronal disruption of Insulin signaling in adult Drosophila reveals no significant defects**.**  **a)** No significant defects are seen for shock reactivity with the expression of chicoRNAi [Elav>chicoRNAi^7776^ or Elav>chicoRNAi^7777^] or UAS-chico [Elav>UAS-chico]. (ANOVA P=0.8963; N=6 PI per genotype) **b)** Similarly, no defects in olfaction of either odors (octanol-PIO; methycyclohexanol-PIM) used in the learning and memory assay is observed for flies expressing chicoRNAi^7776^ or UAS-chico pan neuronally. (ANOVA P= 0.88;N=6 PI per genotype). **c)** No defect is observed in shock sensation with pan-neuronal expression of chicoRNAi^7777^ compared to genetic appropriate controls**.** (ANOVA P= 0.6356; N=6 PI per genotype) **d)** No defect is observed in olfaction for the two odors used in the assay for chicoRNAi^7777^ expressing pan-neuronally or their controls. (ANOVA P=0.7706; N=6 PI per genotype). **e)** Similarly, pan-neuronal expression of InR RNAi [Elav>InRRNAi ^992^ ] or dominant negative [Elav>InR^DN^] does not affect shock reactivity significantly**.** (ANOVA P= 0.1638; N= 6 PI per genotype.**f)** Pan-neuronal expression of InR RNAi or dominant negative does not affect olfaction for the odors used in the assay (ANOVA P=0.6709; N= 6 PI per genotype). **g)** No mushroom body crossing over phenotype is seen with the pan-neuronal epression of chico or InR RNAi used for the behavior experiments. **h)** Pan neuronal expression of UAS-chico leads to lack of normal S6K signal in the central complex and large punctae throughout the brain.

**Supplementary Figure 2.** Normal sensory controls in mushroom body and central complex expressing transgenic flies. **a)** Normal olfaction in flies expressing chicoRNAi ([OK>chicoRNAi^7776^] , [OK>chicoRNAi^7777^]) UAS-chico [OK>UAS-chico] or InR RNAi [OK>InRRNAI^992^] and InR^DN^ [OK>InR^DN^] in mushroom bodies (ANOVA P=0.4255, N=4 PI per genotype). **b)** Normal shock sensitivity in flies expressing chicoRNAi ([OK>chicoRNAi^7776^] , [OK>chicoRNAi^7777^]) UAS-chico [OK>UAS-chico] or InR RNAi [OK>InRRNAI^992^] and InR^DN^ [OK>InR^DN^] in the mushroom bodies (ANOVA P=0.9918, N=4 PI per genotype). **c)** Normal olfaction in flies expressing chicoRNAi ([Feb>chicoRNAi^7776^] , [Feb>chicoRNAi^7777^]) UAS-chico [Feb>UAS-chico] or InR RNAi [Feb>InRRNAI^992^] and InR^DN^ [Feb>InR^DN^] in the central complex (ANOVA P>0.9999, N=4 PI per genotype). **d)** Normal shock responsiveness is observed in FEB170GAL4 expressing expressing chicoRNAi ([Feb>chicoRNAi^7776^] , [Feb>chicoRNAi^7777^]) UAS-chico [Feb>UAS-chico] or InR RNAi [Feb>InRRNAI^992^] and InR^DN^ [Feb>InR^DN^] in the central complex when compared to WT (ANOVA P=0.9614, N=2-4 PI per genotype). All graphs depict mean ± s.e.m.

**Supplementary Figure 3.** Expression of InR or chico RNAi in a fan shape region of the central complex does not impact on olfactory learning or memory. **a)** Expression pattern of 232GAL4 visualized by expression of UAS-mcd8GFP. **b)** 232GAL4 driven expression of chico RNAi [232>ChicoRNAi ^7776^] or InRRNAi [232>InRRNAi^992^] does not result in significant defect in learning. (ANOVA P=0.1086, N= 4 PI per genotype). **c)** Expression of chico RNAi or InR RNAi with 232GAL4 does not result in significant defect in 1 day memory after spaced training (ANOVA P=0.86, N=4PI per genotype. All graphs depict mean ± s.e.m.
